# Supplementary material for: An open-source framework for large-scale, flexible evaluation of biomedical text mining systems
Source: J Biomed Discov Collab. 2008 Jan 29;3:1. doi: 10.1186/1747-5333-3-1 (PMC2276192; doi:10.1186/1747-5333-3-1)
Supplement: Additional file 1 — Evaluation platform source code. The additional file contains the software evaluation framework discussed in this paper. The most current release can be downloaded from SourceForge.net [1]. See the accompanying README file for instructions on installation and use. Also included in the distribution is a collection of UIMA wrappers for some commonly used BioNLP tools and annotated corpora. [file 1747-5333-3-1-S1.zip › bionlp_uima_repository-1.0.1/docs/api/edu/uchsc/ccp/uima/annotation/CCPAnnotationSet.html]

CCPAnnotationSet (Center for Computational Pharmacology Util/UIMA API)


|  |  |  |
| --- | --- | --- |
| **Overview** \| **Package** \| **Class** \| **Tree** \| **Deprecated** \| **Index** \| **Help** | |  |
| **PREV CLASS**   **NEXT CLASS** | **FRAMES**    **NO FRAMES**     **All Classes** |  |
| SUMMARY: NESTED \| FIELD \| CONSTR \| METHOD | DETAIL: FIELD \| CONSTR \| METHOD |  |


---


## edu.uchsc.ccp.uima.annotation Class CCPAnnotationSet

```
java.lang.Object
  org.apache.uima.cas.impl.FeatureStructureImpl
      org.apache.uima.jcas.cas.TOP
          edu.uchsc.ccp.uima.annotation.CCPAnnotationSet
```

**All Implemented Interfaces:**: Cloneable, org.apache.uima.cas.FeatureStructure

---

``` public class CCPAnnotationSet extends org.apache.uima.jcas.cas.TOP ```

The annotation set provides a means for arbitrarily categorizing or clustering groups of annotations. Annotations can be associated with multiple annotation groups. Examples of use include, defining Gold Standard annotation sets, and delineating between the use of different parameters during annotation, among others. Each annotation set is associated with a unique ID, a name and a description.
Updated by JCasGen Tue Aug 28 16:44:10 MDT 2007
XML source: /home/williamb/eclipse/RELEASED\_SOURCE\_CODE/descriptors/typeSystem/CCPTypeSystem.xml

---

| **Field Summary** | |
| --- | --- |
| `static int` | `type` |
| `static int` | `typeIndexID` |

**Fields inherited from class org.apache.uima.jcas.cas.TOP**

`addr, jcasType`


| **Constructor Summary** | |
| --- | --- |
| `protected` | `CCPAnnotationSet()`             Never called. |
|  | `CCPAnnotationSet(int addr, org.apache.uima.jcas.cas.TOP_Type type)`             Internal - constructor used by generator |
|  | `CCPAnnotationSet(org.apache.uima.jcas.JCas jcas)` |


| **Method Summary** | |
| --- | --- |
| `String` | `getAnnotationSetDescription()`             getter for annotationSetDescription - gets A textual description of an annotation set. |
| `int` | `getAnnotationSetID()`             getter for annotationSetID - gets An integer uniquely identifying a particular annotation set. |
| `String` | `getAnnotationSetName()`             getter for annotationSetName - gets The name of the annotation set. |
| `int` | `getTypeIndexID()` |
| `void` | `setAnnotationSetDescription(String v)`             setter for annotationSetDescription - sets A textual description of an annotation set. |
| `void` | `setAnnotationSetID(int v)`             setter for annotationSetID - sets An integer uniquely identifying a particular annotation set. |
| `void` | `setAnnotationSetName(String v)`             setter for annotationSetName - sets The name of the annotation set. |

**Methods inherited from class org.apache.uima.jcas.cas.TOP**

`addToIndexes, addToIndexes, equals, getAddress, getCAS, getCASImpl, getLowLevelCas, hashCode, removeFromIndexes, removeFromIndexes`

**Methods inherited from class org.apache.uima.cas.impl.FeatureStructureImpl**

`clone, getBooleanValue, getByteValue, getDoubleValue, getFeatureValue, getFeatureValueAsString, getFloatValue, getIntValue, getLongValue, getShortValue, getStringValue, getType, prettyPrint, prettyPrint, prettyPrint, setBooleanValue, setByteValue, setDoubleValue, setFeatureValue, setFeatureValueFromString, setFloatValue, setIntValue, setLongValue, setShortValue, setStringValue, toString, toString`

**Methods inherited from class java.lang.Object**

`finalize, getClass, notify, notifyAll, wait, wait, wait`

**Field Detail**

### typeIndexID

```
public static final int typeIndexID
```

---


### type

```
public static final int type
```


**Constructor Detail**

### CCPAnnotationSet

```
protected CCPAnnotationSet()
```

:   Never called. Disable default constructor

---


### CCPAnnotationSet

```
public CCPAnnotationSet(int addr,
                        org.apache.uima.jcas.cas.TOP_Type type)
```

:   Internal - constructor used by generator

---


### CCPAnnotationSet

```
public CCPAnnotationSet(org.apache.uima.jcas.JCas jcas)
```


**Method Detail**

### getTypeIndexID

```
public int getTypeIndexID()
```

:   **Overrides:**: `getTypeIndexID` in class `org.apache.uima.jcas.cas.TOP`

---


### getAnnotationSetID

```
public int getAnnotationSetID()
```

:   getter for annotationSetID - gets An integer uniquely identifying a particular annotation set.

---


### setAnnotationSetID

```
public void setAnnotationSetID(int v)
```

:   setter for annotationSetID - sets An integer uniquely identifying a particular annotation set.

---


### getAnnotationSetName

```
public String getAnnotationSetName()
```

:   getter for annotationSetName - gets The name of the annotation set.

---


### setAnnotationSetName

```
public void setAnnotationSetName(String v)
```

:   setter for annotationSetName - sets The name of the annotation set.

---


### getAnnotationSetDescription

```
public String getAnnotationSetDescription()
```

:   getter for annotationSetDescription - gets A textual description of an annotation set.

---


### setAnnotationSetDescription

```
public void setAnnotationSetDescription(String v)
```

:   setter for annotationSetDescription - sets A textual description of an annotation set.


---


|  |  |  |
| --- | --- | --- |
| **Overview** \| **Package** \| **Class** \| **Tree** \| **Deprecated** \| **Index** \| **Help** | |  |
| **PREV CLASS**   **NEXT CLASS** | **FRAMES**    **NO FRAMES**     **All Classes** |  |
| SUMMARY: NESTED \| FIELD \| CONSTR \| METHOD | DETAIL: FIELD \| CONSTR \| METHOD |  |


---
